# New Shape Function for the Bending Analysis of Functionally Graded Plate

**DOI:** 10.3390/ma11122381

**Published:** 2018-11-26

**Authors:** Dragan Čukanović, Aleksandar Radaković, Gordana Bogdanović, Milivoje Milanović, Halit Redžović, Danilo Dragović

**Affiliations:** 1 Faculty of Technical Sciences, University of Priština, 38220 Kosovska Mitrovica, Serbia; dragan.cukanovic@pr.ac.rs; 2 Department of Technical Sciences, State University of Novi Pazar, 36300 Novi Pazar, Serbia; milanovicnp@gmail.com (M.M.); halit.redzovic@gmail.com (H.R.); danilo.dragovic@yahoo.com (D.D.); 3 Faculty of Engineering, University of Kragujevac, 34000 Kragujevac, Serbia; gocab@kg.ac.rs

**Keywords:** functionally graded plate, power-law distribution, high order shear deformation theory, elastic foundation

## Abstract

The bending analysis of thick and moderately thick functionally graded square and rectangular plates as well as plates on Winkler–Pasternak elastic foundation subjected to sinusoidal transverse load is presented in this paper. The plates are assumed to have isotropic, two-constituent material distribution through the thickness, and the modulus of elasticity of the plate is assumed to vary according to a power-law distribution in terms of the volume fractions of the constituents. This paper presents the methodology of the application of the high order shear deformation theory based on the shape functions. A new shape function has been developed and the obtained results are compared to the results obtained with 13 different shape functions presented in the literature. Also, the validity and accuracy of the developed theory was verified by comparing those results with the results obtained using the third order shear deformation theory and 3D theories. In order to determine the procedure for the analysis and the prediction of behavior of functionally graded plates, the new program code in the software package MATLAB has been developed based on the theories studied in this paper. The effects of transversal shear deformation, side-to-thickness ratio, and volume fraction distributions are studied and appropriate conclusions are given.

## 1. Introduction

Failure and delamination at the border between two layers are the biggest and the most frequently studied problem of the conventional composite laminates. Delamination of layers due to high local inter-laminar stresses causes a reduction of stiffness and a loss of structural integrity of a construction. In order to eliminate these problems, improved materials such as functionally graded materials (FGM), which are getting more and more popular, are used for innovative engineering constructions.

**Figure 1:**
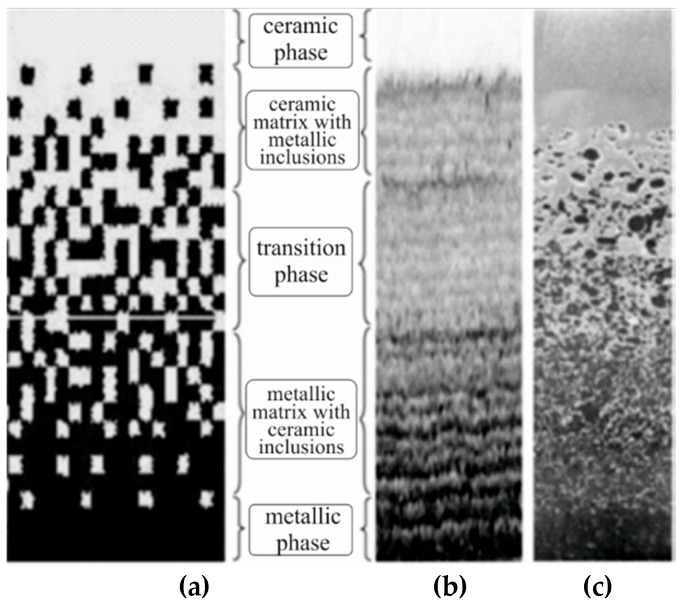
Schematic of continuously graded microstructure with metal-ceramic constituents: (**a**) smoothly graded microstructure; (**b**) enlarged view; (**c**) ceramic-metal functionally graded materials (FGM).

FGM is a composite material consisting of two or more constituents with the continuous change of properties in a certain direction. In other words, these materials can also be defined as materials which possess a gradient change of properties due to material heterogeneity. A gradient property can go in one or more directions and it can also be continuous or discontinuous from one surface to another depending on the production technique [1,2,3]. One of the most common uses of FGM materials is found in thermal barriers, one surface of which is in contact with high temperatures and is made of ceramic which can provide adequate thermal stability, low thermal conductivity, and fine antioxidant properties. The low-temperature side of the barrier is made of metal, which is superior in terms of mechanical strength, toughness, and high thermal conductivity. Functionally graded materials, which contain metal and ceramic constituents, improve thermo-mechanical properties between layers, because of which delamination of layers should be avoided due to continuous change between properties of the constituents. By varying the percentage of volume fraction content of the two or more materials, FGM can be formed so that it achieves a desired gradient property in specific directions. Figure 1 shows schematic of continuously graded microstructure with metal-ceramic constituents [4].

**Figure 2:**
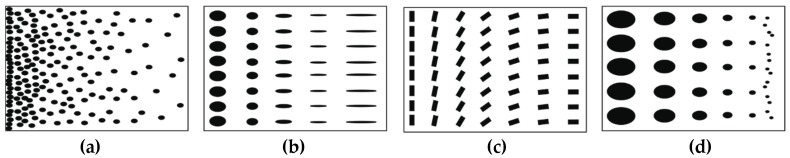
Different types of functionally graded materials based on nature of gradients: (**а**) fraction gradient type; (**b**) shape gradient type; (**c**) orientation gradient type; (**d**) size gradient type.

Depending on the nature of gradient, functionally graded materials may be grouped into fraction gradient type, shape gradient type, orientation gradient type and size gradient type (Figure 2) [5].

With the expansion of the FGM material application area, it was necessary to improve fabrication methods for mentioned materials. Various fabrication methods have been developed for the preparation of bulk FGMs and graded thin films. The processing methods are commonly classified into four groups like powder technology methods (dry powder processing, slip vesting, tape casting, infiltration process or electrochemical gradation, powder injection molding and self-propagating high temperature synthesis, etc.), deposition methods (chemical vapor deposition, physical vapor deposition, electrophoretic deposition, slurry deposition, pulsed laser deposition, plasma spraying, etc.), in-situ processing methods (laser cladding, spray forming, sedimentation and solidification, centrifugal casting, etc.), and rapid prototyping processes (multiphase jet solidification, 3D-printing, laser printing, laser sintering, etc.) [6]. The basic difference between the mentioned production methods can be made according to whether the obtained materials have a stepwise or continuous structure. The main disadvantage of the methods based on powder metallurgy is that it is very difficult to obtain FGM with a continuous change in properties. Continuous graded structures are produced by methods based on casting. Taking this fact into consideration, it was necessary to develop functions which would, with a smaller degree of approximation and in the best way possible, describe a gradient change of properties in a desired direction [7,8].

The majority of already existing software for the analysis of the composite materials are based on the classical plate theory (CPT) [9] and first-order shear deformation theories (FSDT), which were developed by Mindlin [10] and, in a similar way, by Reissner [11,12]. Although classical theory does not consider the effect of transverse shear stresses, it can provide acceptable predictions of the behavior and the results for thin FGM plates where the effects of shear and normal strains across the thickness of the plate are negligible.

Static problems of buckling and bending of FGM plates by using the CPT for different cases of boundary conditions were studied by the authors of the following papers [13,14,15]. Considering von Karman’s type of geometric nonlinearity, FGM behavior was analyzed in [16,17]. The effect of a gradient distribution of materials in thin square and rectangular FGM plates was studied in terms of different cases of dynamic load. The papers [18,19] analyze free vibrations of the FGM plates using CPT for different boundary conditions in the area of geometric linearity. Von Karman’s type of nonlinearity has been used in the papers [20,21].

Mindlin’s and Reissner’s theories take into consideration the effect of shear stresses across the thickness of the plate and require the use of correctional factors which generally depend on the shape and geometry. FSDT theory has been widely used in numerous papers mainly for solving nonlinear problems [22,23]. Static problems due to introducing geometric nonlinearity have been studied in [24], using Green’s strain tensor, and in [25] using von Karman’s strain tensor.

In order to avoid the use of shear correctional factors, high-order shear deformation theories (HSDT) have been introduced. HSDT theories can be developed by developing displacement components into power series at the coordinate of thickness. Generally, in the theories developed in this way, desired precision of the analysis can be achieved by introducing a sufficient number of terms in the power series. The most frequently used HSDT theory is the third-order shear deformation theory (TSDT) developed for composite laminates [26,27], which takes into consideration the effects of shear strains by satisfying the condition of keeping the upper and lower surface of the laminates free of stresses. Later, that theory was used in the analysis of FGM plates [28,29] for solving buckling problems [30,31], free vibrations and dynamic stability [32,33]. In addition to TSDT, there are HSDT theories based on the shape functions which represent a special group of HSDT theories introduced in order to eliminate the need for correctional factors [34,35]. Contrary to CPT and FSDT, the supposed displacement shapes in this theory do not foresee that the normal to the middle plane of the laminate plate remain a straight line, but that during deformation the normal will become curved. Generally, shape functions can be polynomial, hyperbolic, exponential, parabolic etc. Polynomial HSDTs usually diverge from other types of these theories and in accordance to the order of a polynomial at the thickness coordinate they are categorized into the group of second-order shear deformation theories (SSDT) or third-order shear deformation theories. Polynomial theories are those that are most common in the articles, which deal with FG plates’ analysis using HSDT. According to [36,37] all polynomial HSDT of third order can be classified so that the supposed displacement fields contain eleven unknowns. The above-mentioned formulation has been expanded in [38,39] by supposing that the displacements are cubic functions of the thickness coordinate of the plate, that is, the supposed displacements contain twelve independent variables. In [40,41,42] the authors have proposed a shear deformation theory of n-series, which was obtained by modifying the displacement field of TSDT, in order to explain polynomial elements of n-series. Unlike HSDT based on polynomial shape functions, some authors have dealt with researching and introducing different hyperbolic, exponential, parabolic, and other shape functions [43,44,45,46,47,48,49,50,51]. Proposed functions were applied in the analysis of conventional laminate composites with the aim to describe the behavior of moderately thick and thick under different static and dynamic loads.

On the other hand, continuum-based 3D elasticity theory could be used for the analysis of these plates. However, 3D solution methods are mathematically complex which consequently results in prolonged calculation time and the need for high performance hardware. Taking the aforementioned into consideration, developing and using 2D shear deformation plate theories, which consider the effects of previously mentioned shear and normal strains and provide the precision in the same way as 3D models do, represents a trend in the process of analysis of FGM plates.

This paper presents, in detail, the methodology of the application of the HSDT theory based on the shape functions. A new shape function has been developed and the obtained results are compared to the results obtained with 13 different shape functions presented in the papers from the reference list. Also, the results have been verified through comparison with the results obtained with TSDT and 3D theories. In order to determine a procedure for the analysis and the prediction of behavior of FGM plates, the new program code in the software package MATLAB (MATrix LABoratory) has been developed based on theories studied in this paper.

Finally, the ultimate goal and the purpose of all the previously mentioned studies and analyses is the application of FGM in different areas of engineering and branches of industry. Although FGM were initially used as materials for thermal barrier in space shuttles, today they are becoming widely used in the field of medicine, dentistry, energy and nuclear sector, automotive industry, military, optoelectronics etc.

## 2. Description of the Problem

**Figure 3:**
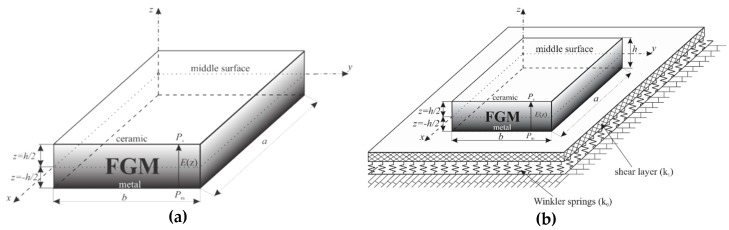
Geometry of the plate: (**a**) FGM plate; (**b**) FGM plate on elastic foundation.

The subject of the analysis in this paper are FGM plate (Figure 3a) and FGM plate on elastic foundation (Figure 3b). The plate (length *a*, width *b* and height *h*) is made of functionally graded material consisting of the two constituents, namely, metal and ceramics.

**Figure 4:**
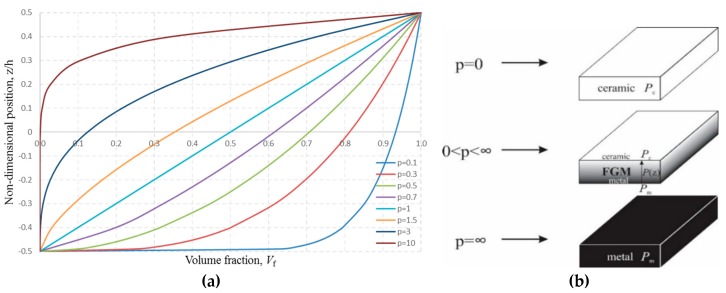
The comparison of homogenous plates (ceramic or metal) and FGM plates: (**a**) volume fraction of the material; (**b**) homogenous and FGM plates.

It is assumed that mechanical properties of the FGM in the thickness direction of the plate change according to the power law distribution (Figure 4a):

(1) $$P\left(z\right)=P_{m}+P_{\mathrm{cm}}{\left(\frac{1}{2}+\frac{z}{h}\right)}^{p},\;P_{\mathrm{cm}}=P_{c}-P_{m}.$$

This law defines the change of the mechanical properties as the function of the volume fraction of the FGM constituents in the thickness direction of the plate.

In the Equation (1), *h* represents total thickness of the plate, and *P*(*z*) represents a material property in an arbitrary cross-section *z*, −*h*/2 < *z* < *h*/2. *P*_c_ represents the material property at the top of the plate *z* = *h*/2 − ceramic, and *P*_m_ represents the material property at the bottom of the plate *z* = −*h*/2 − metal. Index *p* is the exponent of the equation which defines the volume fraction of the constituents in FGM. Practically, by varying the index *p*, homogenous plates as well as FGM plate with precisely determined gradient structure could be obtained, as it is presented in Figure 4b:

- when *p* = 0 the plate is homogenous, made of ceramics,
- when 0 < *p* < ∞ the plate has a gradient structure,
- theoretically, when *p* = ∞ the plate becomes homogenous again, made of metal, although the plate can be considered homogenous even when *p* > 20.

## 3. Kinematic Displacement-Strain Relations and Constitutive Equation of Elasticity for FGM

According to HSDT based on the shape functions, displacements could be presented in the following way:

(2) $$\begin{array}{l} u\left(x,y,z,t\right)=u_{0}\left(x,y,t\right)-z\frac{\partial w_{0}\left(x,y,t\right)}{\partial x}+f\left(z\right)\theta_{x},\\ v\left(x,y,z,t\right)=v_{0}\left(x,y,t\right)-z\frac{\partial w_{0}\left(x,y,t\right)}{\partial y}+f\left(z\right)\theta_{y},\\ w\left(x,y,z,t\right)=w_{0}\left(x,y,t\right), \end{array}$$

where: $u_{0},\;v_{0},\;w_{0}$ are displacement components in the middle plane of the plate, $\frac{\partial w_{0}}{\partial x},\;\frac{\partial w_{0}}{\partial y}$ are rotation angles of transverse normal in relation to *x* and *y* axes, respectively, $\theta_{x},\;\theta_{y}$ are rotations of the transverse normal due to transverse shear and f(z) is the shape function.

In the reference literature there are many shape functions which can be polynomial, trigonometric, exponential, hyperbolic. Some examples of the shape functions are given in Table 1.

This paper proposes a new shape function as follows:

(3) $$f\left(z\right)=z\left(\cosh\left(\frac{z}{h}\right)-1.388\right)$$

**Figure 5:**
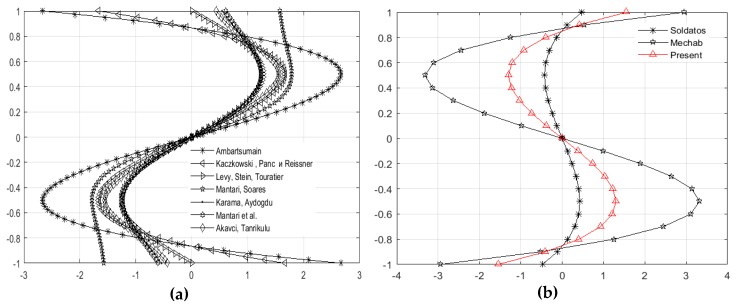
Shape function diagrams: (**a**) shape function from literature; (**b**) new proposed shape function.

The introduced shape function is an odd function of the thickness coordinate z and satisfies zero stress conditions for out of plane shear stresses. Observing the shape functions in the Table 1, may see that the proposed function belongs to the group of simple mathematic functions. This fact makes the integration process easier and thus reduces considerably the calculation time. Having in mind that the function is analytically integrable, there is no need to switch to numeric integration, which additionally increases the precision of the obtained results. The verification of the above claims is shown in the comparative diagrams (Figure 5) of the newly introduced shape function and the shape functions given in the Table 1. These shape functions’ diagrams can be categorized into two groups of functions. In both cases it can be seen in the diagram that, in the case of the ratio *z*/*h* = 0.5, all shape functions have extreme values, which are different (Figure 5a). The proposed new shape function (3) belongs to the second group (Figure 5b), together with the functions of Soldatos and Mechab which are also analytically integrable functions.

For small displacements and moderate rotations of a transverse normal in relation to *x* axis and *y* axis, normal and shear strain components are obtained by well-known relations in linear elasticity between displacements and strains:

(4) $$\varepsilon =\varepsilon^{\left(0\right)}+zk^{\left(0\right)}+f(z)k^{\left(1\right)},\;\gamma =f^{\prime }(z)k^{\left(2\right)},$$

where:

(5) $$\begin{array}{l} \varepsilon =\left\{\begin{array}{c} \varepsilon_{xx}\\ \varepsilon_{yy}\\ \gamma_{xy} \end{array}\right\},\;\gamma =\left\{\begin{array}{c} \gamma_{xz}\\ \gamma_{yz} \end{array}\right\},\;\varepsilon^{\left(0\right)}=\left\{\begin{array}{c} \varepsilon_{xx}^{\left(0\right)}\\ \varepsilon_{yy}^{\left(0\right)}\\ \gamma_{xy}^{\left(0\right)} \end{array}\right\}=\left\{\begin{array}{c} u_{0,x}\\ v_{0,y}\\ u_{0,y}+v_{0,x} \end{array}\right\},\;\\ k^{\left(0\right)}=\left\{\begin{array}{c} k_{xx}^{\left(0\right)}\\ k_{yy}^{\left(0\right)}\\ k_{xy}^{\left(0\right)} \end{array}\right\}=\left\{\begin{array}{c} -w_{0,xx}\\ -w_{0,yy}\\ -2w_{0,xy} \end{array}\right\},\;k^{\left(1\right)}=\left\{\begin{array}{c} k_{xx}^{\left(1\right)}\\ k_{yy}^{\left(1\right)}\\ k_{xy}^{\left(1\right)} \end{array}\right\}=\left\{\begin{array}{c} \theta_{x,x}\\ \theta_{y,y}\\ \theta_{x,y}+\theta_{y,x} \end{array}\right\},\;k^{\left(2\right)}=\left\{\begin{array}{c} k_{xz}^{\left(2\right)}\\ k_{yz}^{\left(2\right)} \end{array}\right\}=\left\{\begin{array}{c} \theta_{x}\\ \theta_{y} \end{array}\right\}, \end{array}$$

where $f^{\prime }(z)=\frac{df(z)}{dz}$ is the first derivative of the shape function in the thickness direction of the plate.

The elastic constitutive relations for FGM are given as follows:

(6) $$\left\{\begin{array}{l} \sigma_{xx}\\ \sigma_{yy}\\ \tau_{xz}\\ \tau_{yz}\\ \tau_{xy} \end{array}\right\}=\left[\begin{array}{ccccc} C_{11}\left(z\right) & C_{12}\left(z\right) & 0 & 0 & 0\\ C_{12}\left(z\right) & C_{22}\left(z\right) & 0 & 0 & 0\\ 0 & 0 & C_{44}\left(z\right) & 0 & 0\\ 0 & 0 & 0 & C_{55}\left(z\right) & 0\\ 0 & 0 & 0 & 0 & C_{66}\left(z\right) \end{array}\right]\left\{\begin{array}{l} \varepsilon_{xx}\\ \varepsilon_{yy}\\ \gamma_{xz}\\ \gamma_{yz}\\ \gamma_{xy} \end{array}\right\},$$

where the coefficients of the constitutive elasticity tensor could be defined through engineering constants:

(7) $$C_{11}\left(z\right)=C_{22}\left(z\right)=\frac{E\left(z\right)}{1-\nu^{2}},\;C_{44}\left(z\right)=C_{55}\left(z\right)=C_{66}\left(z\right)=\frac{E\left(z\right)}{2\left(1+\nu \right)},\;C_{12}\left(z\right)=\frac{\nu E\left(z\right)}{1-\nu^{2}}.$$

Due to the gradient change of the plate structure in the direction of the *z* coordinate, based on (1), the modulus of elasticity could be defined as:

(8) $$E\left(z\right)=E_{m}+E_{\mathrm{cm}}{\left(\frac{1}{2}+\frac{z}{h}\right)}^{p},\;E_{\mathrm{cm}}=E_{c}-E_{m},$$

while Poisson’s ratio ν is considered constant due to a small value variation in the thickness direction of the plate, ν=const.

As it could be seen, the coefficients of the constitutive tensor are functionally dependent on the *z* coordinate which practically means that for p≠0 there is a finite number of planes parallel to the middle plane, where each of these planes has different values of the constitutive tensor $C_{ij}$.

## 4. Bending of FGM Plates and FGM Plates on Elastic Foundation

It is assumed that the plate is loaded with an arbitrary transverse load q(x,y). Work under external load is defined as:

(9) $$V=-\frac{1}{2}\int_{A}qwdA,$$

where:

(10) $$q(x,y)=q_{0}\sin\left(\frac{\pi x}{a}\right)\sin\left(\frac{\pi y}{b}\right),$$

is the sinusoidal transverse load with an amplitude $q_{0}$.

Plate strain energy is defined as:

(11) $$\begin{array}{ll} U & =\int_{A}(N_{xx}\varepsilon_{xx}^{\left(0\right)}+N_{yy}\varepsilon_{yy}^{\left(0\right)}+N_{xy}\gamma_{xy}^{\left(0\right)}+M_{xx}k_{xx}^{\left(0\right)}+M_{yy}k_{yy}^{\left(0\right)}+M_{xy}k_{xy}^{\left(0\right)}\\ & +\;P_{xx}k_{xx}^{\left(1\right)}+P_{yy}k_{yy}^{\left(1\right)}+P_{xy}k_{xy}^{\left(1\right)}+R_{x}k_{xz}^{\left(2\right)}+R_{y}k_{yz}^{\left(2\right)})dA, \end{array}$$

where force, moments and higher order moments vectors are obtained in the following form:

(12) $$\begin{array}{l} N=\int_{-h/2}^{h/2}\sigma dz=\int_{-h/2}^{h/2}C_{P}\varepsilon dz=\int_{-h/2}^{h/2}C_{P}\varepsilon^{\left(0\right)}dz+\int_{-h/2}^{h/2}C_{P}k^{\left(0\right)}zdz+\int_{-h/2}^{h/2}C_{P}k^{\left(1\right)}f\left(z\right)dz,\\ M=\int_{-h/2}^{h/2}\sigma zdz=\int_{-h/2}^{h/2}C_{P}\varepsilon zdz=\int_{-h/2}^{h/2}C_{P}\varepsilon^{\left(0\right)}zdz+\int_{-h/2}^{h/2}C_{P}k^{\left(0\right)}z^{2}dz+\int_{-h/2}^{h/2}C_{P}k^{\left(1\right)}zf\left(z\right)dz,\\ P=\int_{-h/2}^{h/2}\sigma f\left(z\right)dz=\int_{-h/2}^{h/2}C_{P}\varepsilon f\left(z\right)dz=\int_{-h/2}^{h/2}C_{P}\varepsilon^{\left(0\right)}f\left(z\right)dz+\int_{-h/2}^{h/2}C_{P}k^{\left(0\right)}zf\left(z\right)dz+\int_{-h/2}^{h/2}C_{P}k^{\left(1\right)}{\left(f\left(z\right)\right)}^{2}dz,\\ R=\int_{-h/2}^{h/2}\tau f^{\prime }\left(z\right)dz=\int_{-h/2}^{h/2}C_{S}k^{\left(2\right)}{\left(f^{\prime }\left(z\right)\right)}^{2}dz, \end{array}$$

Matrices in the developed form could be presented in the following way:

(13) $$\begin{array}{l} N=\left\{\begin{array}{c} N_{xx}\\ N_{yy}\\ N_{xy} \end{array}\right\},\;M=\left\{\begin{array}{c} M_{xx}\\ M_{yy}\\ M_{xy} \end{array}\right\},\;P=\left\{\begin{array}{c} P_{xx}\\ P_{yy}\\ P_{xy} \end{array}\right\},\;R=\left\{\begin{array}{c} R_{x}\\ R_{y} \end{array}\right\},\\ C_{P}=\left[\begin{array}{ccc} C_{11} & C_{12} & 0\\ C_{12} & C_{22} & 0\\ 0 & 0 & {\bar{C}}_{66} \end{array}\right],\;C_{S}=\left[\begin{array}{cc} C_{44} & 0\\ 0 & C_{55} \end{array}\right],\;\sigma =\left\{\begin{array}{c} \sigma_{xx}\\ \sigma_{yy}\\ \tau_{xy} \end{array}\right\},\;\tau =\left\{\begin{array}{c} \tau_{xz}\\ \tau_{yz} \end{array}\right\}. \end{array}$$

In the Equation (12) by grouping the terms with the elements of constitutive tensor, new matrices with the following components could be defined:

(14) $$\begin{array}{lll} A_{ij}=\int_{-h/2}^{h/2}C_{ij}dz, & B_{ij}=\int_{-h/2}^{h/2}C_{ij}zdz, & \\ D_{ij}=\int_{-h/2}^{h/2}C_{ij}f\left(z\right)dz, & E_{ij}=\int_{-h/2}^{h/2}C_{ij}z^{2}dz, & \left(i,\;j\right)=\left(1,\;2,\;6\right),\\ F_{ij}=\int_{-h/2}^{h/2}C_{ij}zf\left(z\right)dz, & G_{ij}=\int_{-h/2}^{h/2}C_{ij}{\left(f\left(z\right)\right)}^{2}dz, & \\ H_{lr}=\int_{h^{-}}^{h^{+}}C_{lr}{\left(f^{\prime }\left(z\right)\right)}^{2}dz, & & \left(l,\;r\right)=\left(4,\;5\right), \end{array}$$

Therefore, load vectors could now be defined in the following form:

(15) $$\left\{\begin{array}{c} N\\ M\\ P \end{array}\right\}=\left[\begin{array}{ccc} A_{ij} & B_{ij} & D_{ij}\\ B_{ij} & E_{ij} & F_{ij}\\ D_{ij} & F_{ij} & G_{ij} \end{array}\right]\left\{\begin{array}{c} \varepsilon^{\left(0\right)}\\ k^{\left(0\right)}\\ k^{\left(1\right)} \end{array}\right\},\;\left\{R\right\}=\left[H_{lr}\right]\left\{k^{\left(2\right)}\right\},$$

By exchanging plate strain energy (11) and work under external load (9) into the equation which defines the minimum total potential energy principle:

(16) δU+δV=δ(U+V)≡δП=0,

The following form is obtained:

(17) $$\begin{array}{ll} \delta П & =\int_{A}(N_{xx}\delta \varepsilon_{xx}^{\left(0\right)}+N_{yy}\delta \varepsilon_{yy}^{\left(0\right)}+N_{xy}\delta \gamma_{xy}^{\left(0\right)}+M_{xx}\delta k_{xx}^{\left(0\right)}+M_{yy}\delta k_{yy}^{\left(0\right)}+M_{xy}\delta k_{xy}^{\left(0\right)}\\ & +P_{xx}\delta k_{xx}^{\left(1\right)}+P_{yy}\delta k_{yy}^{\left(1\right)}+P_{xy}\delta k_{xy}^{\left(1\right)}+R_{x}\delta k_{xz}^{\left(2\right)}+R_{y}\delta k_{yz}^{\left(2\right)})dA-\int_{A}q\delta wdA=0. \end{array}$$

By exchanging the strain components (5) and by applying the calculus of variations, the following equilibrium equations are obtained:

(18) $$\begin{array}{l} \delta u_{0}:\;N_{xx,x}+N_{xy,y}=0,\\ \delta v_{0}:\;N_{yy,y}+N_{xy,x}=0,\\ \delta w_{0}:\;M_{xx,xx}+2M_{xy,xy}+M_{yy,yy}+q=0,\\ \delta \theta_{x}:\;P_{xx,x}+P_{xy,y}-R_{x}=0,\\ \delta \theta_{y}:\;P_{xy,x}+P_{yy,y}-R_{y}=0. \end{array}$$

which could be further solved through analytical and numerical methods.

In the case of a plate on elastic foundation, in the Equation (16) deformation energy of the elastic foundation should be taken into consideration, which is defined using Winkler–Pasternak model in the following way:

(19) $$U_{e}=\frac{1}{2}\int_{A}\left\{k_{0}w^{2}+k_{1}\left[{\left(\frac{\partial w}{\partial x}\right)}^{2}+{\left(\frac{\partial w}{\partial x}\right)}^{2}\right]\right\}dA.$$

Using the previously mentioned the minimum total potential energy principle, the equilibrium equations of the plate on elastic foundation are the following:

(20) $$\begin{array}{l} \delta u_{0}:\;N_{xx,x}+N_{xy,y}=0,\\ \delta v_{0}:\;N_{yy,y}+N_{xy,x}=0,\\ \delta w_{0}:\;M_{xx,xx}+2M_{xy,xy}+M_{yy,yy}+N_{xx}w_{0,xx}+2N_{xy}w_{0,xy}+N_{yy}w_{0,yy}\\ \;+q-k_{0}w_{0}+k_{1}\left(w_{0,xx}+w_{0,yy}\right)=0,\\ \delta \theta_{x}:\;P_{xx,x}+P_{xy,y}-R_{x}=0,\\ \delta \theta_{y}:\;P_{xy,x}+P_{yy,y}-R_{y}=0. \end{array}$$

## 5. Analytical Solution of the Equilibrium Equations

Although analytical solution methods are limited to simple geometrical problems, boundary conditions and loads, they can provide a clear understanding of the physical aspect of the problem and its solutions are very precise. Since analytical solutions are extremely important for developing new theoretical models, primarily due to their understanding of the physical aspects of the problem, and considering that a new HSDT theory based on a new shape function has been developed in this paper, the analytical solution of the equilibrium equations for a rectangular plate will be presented in the following part of the paper. For complex engineering calculations, which include solving the system of a large number of equations, it is necessary to use numerical methods which provide approximate, but satisfactory results.

For a simply supported rectangular FGM plate, boundary conditions are defined based on [57] as:

(21) $$\begin{array}{l} v_{0}=w_{0}=\theta_{y}=N_{xx}=M_{xx}=P_{xx}=0,\;\mathrm{on the edges where}\;x=0\;\mathrm{or}\;x=a,\\ u_{0}=w_{0}=\theta_{x}=N_{yy}=M_{yy}=P_{yy}=0,\;\mathrm{on the edges where}\;y=0\;\mathrm{or}\;y=b. \end{array}$$

In order to satisfy these kinematic boundary conditions, assumed forms of Navier’s solutions are introduced:

(22) $$\begin{array}{l} u_{0}\left(x,y,t\right)=\sum_{m=1}^{\infty }\sum_{n=1}^{\infty }U_{mn}\cos\frac{m\pi x}{a}\sin\frac{n\pi y}{b},\;v_{0}\left(x,y,t\right)=\sum_{m=1}^{\infty }\sum_{n=1}^{\infty }V_{mn}\sin\frac{m\pi x}{a}\cos\frac{n\pi y}{b},\\ w_{0}\left(x,y,t\right)=\sum_{m=1}^{\infty }\sum_{n=1}^{\infty }W_{mn}\sin\frac{m\pi x}{a}\sin\frac{n\pi y}{b},\\ \theta_{x}\left(x,y,t\right)=\sum_{m=1}^{\infty }\sum_{n=1}^{\infty }T_{xmn}\cos\frac{m\pi x}{a}\sin\frac{n\pi y}{b},\;\theta_{y}\left(x,y,t\right)=\sum_{m=1}^{\infty }\sum_{n=1}^{\infty }T_{ymn}\sin\frac{m\pi x}{a}\cos\frac{n\pi y}{b}. \end{array}$$

The equilibrium equation is further developed into:

(23) $$\underset{L}{\underset{︸}{\left[\begin{array}{ccccc} L_{11} & L_{12} & L_{13} & L_{14} & L_{15}\\ L_{12} & L_{22} & L_{23} & L_{24} & L_{25}\\ L_{13} & L_{23} & L_{33} & L_{34} & L_{35}\\ L_{14} & L_{24} & L_{34} & L_{44} & L_{45}\\ L_{15} & L_{25} & L_{35} & L_{45} & L_{55} \end{array}\right]}}\underset{\bar{U}}{\underset{︸}{\left\{\begin{array}{c} U_{mn}\\ V_{mn}\\ W_{mn}\\ T_{xmn}\\ T_{ymn} \end{array}\right\}}}=\underset{P_{p}}{\underset{︸}{\left\{\begin{array}{c} 0\\ 0\\ q_{0}\\ 0\\ 0 \end{array}\right\}}}$$

or:

(24) $$L\bar{U}=P_{P}$$

Through the matrix multiplication of the Equation (24) with $L^{-1}$, the following is obtained:

(25) $$\underset{I}{\underset{︸}{L^{-1}L}}\bar{U}=L^{-1}P_{P}\;\to \;\bar{U}=L^{-1}P_{P}.$$

The Equation (25) fully defines the amplitudes of the assumed displacement components. The displacement components are obtained if the displacement amplitude matrix is multiplied with the vector from trigonometric functions which depend on x and y.

## 6. Numerical Results

In order to apply the previously obtained theoretical results to a simulation of real problems, a new program code for static analysis of FGM plates has been developed within the software package MATLAB. Material properties of the used materials are shown in Table 2 [58].

Normalized values of a vertical displacement $\bar{w}$ (deflection), normal stresses ${\bar{\sigma }}_{xx}$ and ${\bar{\sigma }}_{yy}$, shear stress ${\bar{\tau }}_{xy}$, and transverse shear stresses ${\bar{\tau }}_{xz}$ and ${\bar{\tau }}_{yz}$ are given by using HSDT theory based on the new shape function. Normalization of the aforementioned values has been conducted according to (26) as:

(26) $$\begin{array}{lll} \bar{w}=\frac{10E_{c}h^{3}}{q_{0}a^{4}}w\left(\frac{a}{2},\frac{b}{2}\right), & {\bar{\sigma }}_{xx}(z)=\frac{h}{q_{0}a}\sigma_{xx}\left(\frac{a}{2},\frac{b}{2},z\right), & {\bar{\sigma }}_{yy}(z)=\frac{h}{q_{0}a}\sigma_{yy}\left(\frac{a}{2},\frac{b}{2},z\right),\\ {\bar{\tau }}_{xy}(z)=\frac{h}{q_{0}a}\tau_{xy}\left(0,0,z\right), & {\bar{\tau }}_{xz}(z)=\frac{h}{q_{0}a}\tau_{xz}\left(0,\frac{b}{2},z\right), & {\bar{\tau }}_{yz}(z)=\frac{h}{q_{0}a}\tau_{yz}\left(\frac{a}{2},0,z\right). \end{array}$$

Table 3 shows comparative results of the normalized values of displacement and stresses of square plate for two different ratios of length and thickness of the plate (*a*/*h* = 5 and *a*/*h* = 10) and for different values of the index *p*. Verification of the results obtained in this paper has been conducted by comparing them to the results from the reference papers when *a*/*h* = 10. Based on that, the results when *a*/*h* = 5 are provided for different values of the index *p*, i.e., different volume fraction of the constituents in FGM. Using HSDT theory with the new shape function, the obtained results are compared to the results obtained using 13 different shape functions as well as to the results obtained using quasi 3D theory of elasticity [59] and TSDT theory [58]. The results based on the CPT theory are also presented [60] in order to find certain disadvantages of the theory. Based on the comparative results of displacement and stresses, which are provided in this paper and in previously mentioned theories, it could be seen that there is a match with both TSDT theory and quasi 3D theory of elasticity. On the other hand, it is clearly seen that there are some significant differences in the results obtained by CPT theory, especially related to the stress ${\bar{\sigma }}_{xx}$ which shows that CPT theory does not provide satisfying results in the analysis of thick and moderately thick FGM plates. A comparative review of these results with the results obtained using 13 different shape functions shows that the newly given shape function provides almost identical results. However, since these results are given for the plane on a certain height *z* (for example, stress ${\bar{\sigma }}_{xx}$ on the height of h/3 etc.), a real insight into the values obtained by varying the new function could be offered by presenting stress distribution across the thickness of the plate, which is done through appropriate diagrams.

**Figure 6:**
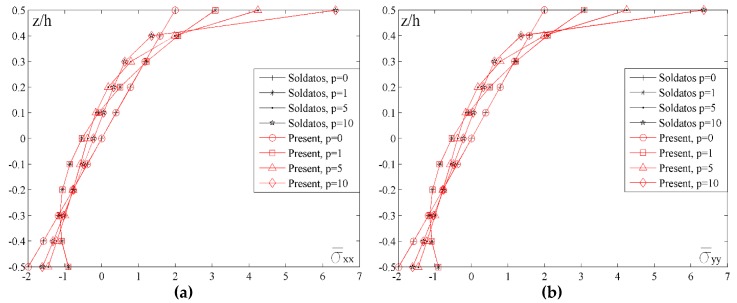
Distribution of the normalized values of the normal stresses ${\bar{\sigma }}_{xx}$ and ${\bar{\sigma }}_{yy}$ across the thickness of the plate for different values of the index *p*: (**a**) *a*/*h* = 10, *a*/*b* = 1; (**b**) *a*/*h* = 10, *a*/*b* = 1.

Figure 6 shows the distribution of normal stresses ${\bar{\sigma }}_{xx}$ and ${\bar{\sigma }}_{yy}$ across the thickness of the plate for different values of the index *p*. By analyzing the diagrams, it could be noticed that the curves representing both stresses are identical. Also, the basic property of FGM could be noticed, namely, the shift of a neutral plane in relation to the plane *z*/*h* = 0. It can also be seen that for the planes at the height *z*/*h* = 0.1–0.15 (depending on the chosen value of the index *p*) normal stresses have a positive sign which clearly indicates extension, and then they change the sign. In case when *p* = 0, (homogenous material made of ceramics) stress distribution is a familiar linear function with the neutral plane when *z*/*h* = 0. Maximum values of normal stresses due to compression are on the lower edge of the plate while the maximum values of normal stresses due to extension are on the upper edge of the plate. It could be noticed that with the increase of the index *p* value, maximum values of stresses due to extension are significantly increased.

**Figure 7:**
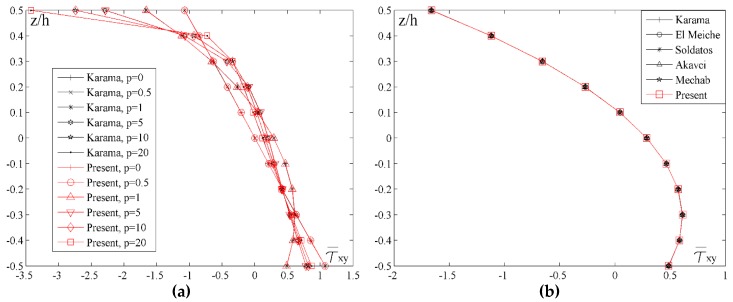
Distribution of the normalized values of the shear stress ${\bar{\tau }}_{xy}$ across the thickness of the plate for different values of the index *p* and different shape function: (**a**) *a*/*h* = 10, *a*/*b* = 1; (**b**) *a*/*h* = 10, *a*/*b* = 1, *p* = 5.

Figure 7 shows the distribution of the shear stress ${\bar{\tau }}_{xy}$ across the thickness of the plate for different values of the index *p* (Figure 7a) and for different shape functions (Figure 7b), but for the unchanging values of *a*/*h* = 10 and *a*/*b* = 1. While analyzing the diagrams, it should be considered that when *p* = 0 the plate is homogenous made of ceramics, when *p* = 20 the plate is homogenous made of metal, and when 0 < *p* < 20 the plate is made of FGM. By analyzing the diagram in the Figure 7a, it could be noticed that for all values of the index *p*, the stress ${\bar{\tau }}_{xy}$ achieves the maximum value on the upper edge of the plate. Ceramic plate has the lowest maximum value. Therefore, with an increase of the metal volume fraction when *p* = 1, maximum stress value also increases and the highest value is achieved when the plate is homogenous made of metal. Moreover, apart from affecting the maximum stress values, the variation of the index *p* value also affects the shape of the ${\bar{\tau }}_{xy}$ stress distribution curve across the thickness of the plate.

In order to conduct a comparative analysis of the results for different shape functions and to estimate the application of the new shape function to the given problems, Figure 7b shows the distribution of the shear stress ${\bar{\tau }}_{xy}$ by using newly developed shape function and the shape functions given in Table 1. It is clearly seen that all the previously mentioned shape functions give identical results to the results obtained with the new shape function.

**Figure 8:**
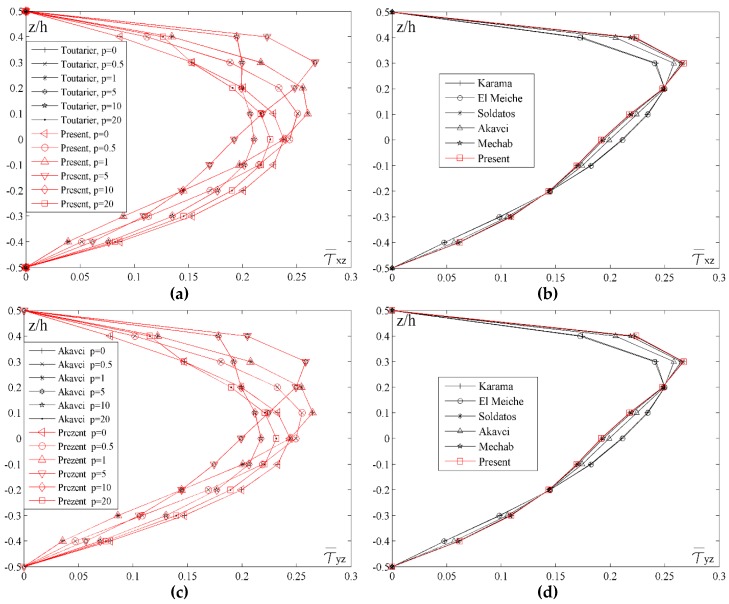
Distribution of the normalized values of the transverse shear stresses ${\bar{\tau }}_{xz}$ and ${\bar{\tau }}_{yz}$ across the thickness of the plate for different values of the index *p* and different shape functions: (**a**) *a*/*h* = 10, *a*/*b* = 1; (**b**) *a*/*h* = 10, *a*/*b* = 1, *p* = 5; (**c**) *a*/*h* = 10, *a*/*b* = 1; (**d**) *a*/*h* = 10, *a*/*b* = 1, *p* = 5.

Figure 8 shows the distribution of transverse shear stresses ${\bar{\tau }}_{xz}$ and ${\bar{\tau }}_{yz}$ across the thickness of the plate for different values of the index *p* and for different shape functions. By analyzing transverse shear stresses in Figure 8a,c, a basic distinction between homogenous and FGM plates can be noticed. When plates are made of ceramics (*p* = 0) or metal (*p* = 20), it can be noticed that both stresses achieve maximum values in the plane at the height *z*/*h* = 0, due to the homogeneity of the material. On the other hand, when FGM plates are considered, there is an asymmetry in relation to the plane *z*/*h* = 0, therefore, when *p* = 1 stresses achieve maximum values in the plane *z*/*h* = 0.15, and when *p* = 5 stresses achieve maximum values when *z*/*h* = 0.3. In contrast to the homogenous ceramic plate, where stress distribution curve is a parabola with the maximum value in the plane *z*/*h* = 0, plates with the larger volume fraction of metal (*p* = 10) also achieve the maximum value of stress when *z*/*h* = 0, but the distribution curve is not a parabola. With the further increase of the metal volume fraction (*p* = 20), and although the plate can be practically considered homogenous, the diagram still shows the curve which is not a parabola. Generally, due to insignificant but still present ceramic fractions in the upper part of the plate, there is a slight deformity of the curve.

By conducting comparative analysis of the stresses ${\bar{\tau }}_{xz}$ and ${\bar{\tau }}_{yz}$ for different shape functions, and with fixed values of *a*/*h* = 10, *a*/*b* = 1 and *p* = 5, it could be seen in Figure 8b,d that, unlike the stress ${\bar{\tau }}_{xy}$, the results do not match for all the shape functions. The most significant deviation could be noticed in the results for the El Meiche’s and Karama’s shape functions. The Akavci’s function also shows a slight deviation and it achieves maximum stress value at the height *z*/*h* = 0.25, while the results for all the other shape functions are almost identical, achieving the maximum stress value in the plane *z*/*h* = 0.25.

**Figure 9:**
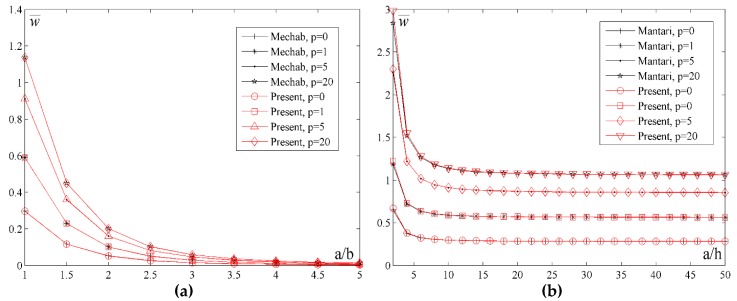
Normalized values of the displacement $\bar{w}$ for different *a*/*h* and *a*/*b* ratios and the values of the index *p*: (**a**) *a*/*h* = 10; (**b**) *a*/*b* = 1.

In order to understand the effects of increasing the index *p* as well as the effect of the thickness and geometry, Figure 9 shows the diagram of the normalized values of the displacement $\bar{w}$ for different *a*/*h* and *a*/*b* ratios and values of the index p. By analyzing Figure 9a,b, it could be noticed that the displacement values $\bar{w}$ for the metal plate (*p* = 20) are the highest, for the ceramic plate they are the lowest, and for the FGM plate they are somewhere in between. Moreover, by varying the volume fraction of metal or ceramics, a desired bending rigidity of the plate could be achieved. In Figure 9a, it could be seen that the curves gradually become closer when *a*/*b* > 4. In contrast to that, Figure 9b shows that with an increase of the ratio *a*/*h* the curves do not become closer, namely, the difference of the displacement ratio remains constant regardless of the index *p* change. This conclusion comes from the fact that in thin plates it is less possible to vary the volume fraction of the FGM constituents in the thickness direction of the plate and, thus, the index *p* has no effects.

In order to determine the effect of the elastic foundation on the displacements and stresses of the FGM plate, the results of different combinations of the FGM constituents have been presented, as well as different combinations of the Winkler (*k*_0_) and Pasternak (*k*_1_) coefficient of the elastic foundation. Apart from the normalization given in (26), it is necessary to apply the normalization of the coefficients *k*_0_ and *k*_1_, in the following form:

(27) $${\bar{k}}_{0}=k_{0}\frac{a^{4}}{D}\;и\;{\bar{k}}_{1}=k_{1}\frac{a^{2}}{D},$$

where the bending stiffness of the plate is $D=\frac{E_{c}h^{3}}{12(1-\nu^{2})}$.

The Table 4 and Table 5 show the results of the normalized values of displacements and stresses of the square plate on elastic foundation for *p* = 5, and *p* = 10, different values of *k*_0_ and *k*_1_ coefficients, as well as for two different ratios length/thickness of the plate (*a*/*h* = 10 and *a*/*h* = 5). In order to determine the effect of the elastic foundation on the displacements and stresses of the plate, the values of displacements and stresses for *k*_0_ = 0 and *k*_1_ = 0 are first shown, which practically matches the case of the plate without the elastic foundation. Afterwards, the values of the given coefficients are varied in order to conclude which of the two has greater influence. Based on the results, it is concluded that the introduction of the coefficient *k*_0_ has less influence on the change of the displacements and stresses values then when only *k*_1_ coefficient is introduced. By introducing *k*_0_ and *k*_1_ coefficients, bending stiffness of the plate increases, i.e., displacement and stresses values decrease and the influence of the Winkler coefficient is smaller than the influence of the Pasternak coefficient. This phenomenon is especially noticeable in the diagram dependency which is to be shown later.

**Figure 10:**
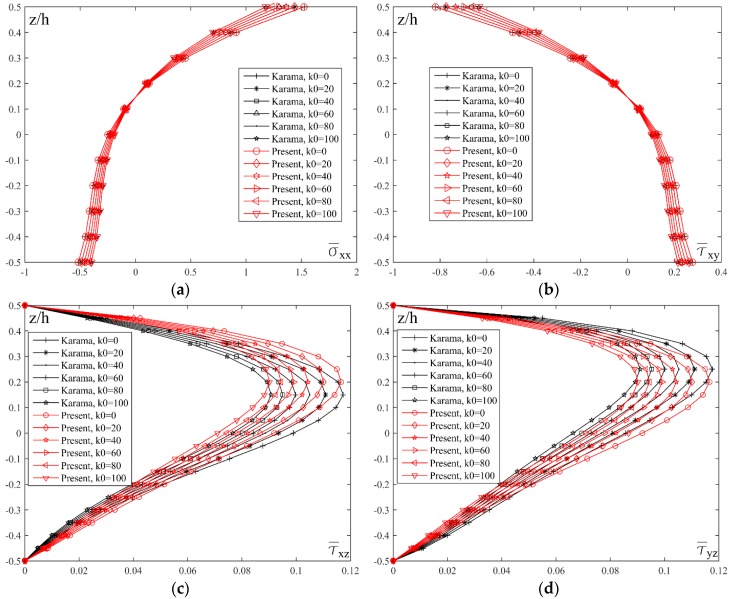
Distribution of the normalized values of the normal stresses ${\bar{\sigma }}_{xx}$, the shear stress ${\bar{\tau }}_{xy}$ and the transverse shear stresses ${\bar{\tau }}_{xz}$ and ${\bar{\tau }}_{yz}$ across the thickness of the plate on elastic foundation for different values of the coefficients *k*_0_: (**a**) *a*/*h* = 10, *a*/*b* = 1, *p* = 2, *k*_1_ = 10; (**b**) *a*/*h* = 10, *a*/*b* = 1, *p* = 2, *k*_1_ = 10; (**c**) *a*/*h* = 10, *a*/*b* = 1, *p* = 2, *k*_1_ = 10; (**d**) *a*/*h* = 10, *a*/*b* = 1, *p* = 2, *k*_1_ = 10.

Figure 10 shows the effect of the Winkler coefficient *k*_0_ on the distribution of the normal stress ${\bar{\sigma }}_{xx}$, shear stress ${\bar{\tau }}_{xy}$ and transversal shear stresses ${\bar{\tau }}_{xz}$ and ${\bar{\tau }}_{yz}$ across the thickness of the plate on the elastic foundation. By analyzing the diagram, it can be seen that the value of the stresses ${\bar{\sigma }}_{xx}$ and ${\bar{\tau }}_{xy}$ equals zero for *z*/*h* = 0.15. On the other hand, the maximum values of ${\bar{\tau }}_{xz}$ and ${\bar{\tau }}_{yz}$ stresses are at *z*/*h* = 0.2 when the new proposed shape function is applied, while the maximum values of mentioned stresses is respectively at *z*/*h* = 0.15 i.e., *z*/*h* = 0.25 for Karama’s shape function.

**Figure 11:**
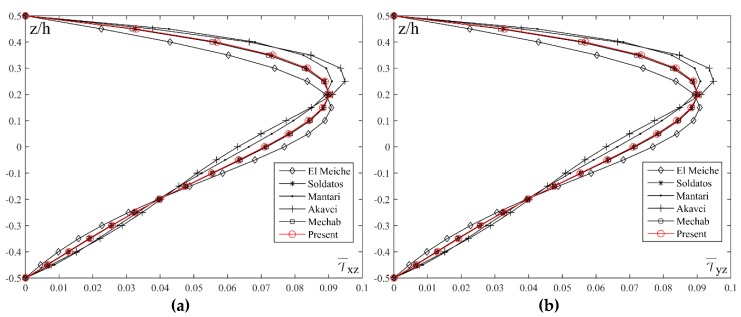
Distribution of the normalized values of the transverse shear stresses ${\bar{\tau }}_{xz}$ and ${\bar{\tau }}_{yz}$ across the thickness of the plate on elastic foundation for different shape functions: (**a**) *a*/*h* = 10, *a*/*b* = 1, *p* = 2, *k*_0_ = 100, *k*_1_ = 10; (**b**) *a*/*h* = 10, *a*/*b* = 1, *p* = 2, *k*_0_ = 100, *k*_1_ = 10.

Figure 11 shows a comparative review of shear transversal stresses ${\bar{\tau }}_{xz}$ and ${\bar{\tau }}_{yz}$ distribution across the thickness of the plate on elastic foundation for different shape functions. As in the case of bending the plate without the elastic foundation, the shape functions do not give the same results. Therefore, it can be seen that for the Mantari’s and Akavci’s shape functions, stresses achieve their maximum values in the plane *z*/*h* = 0.25, and for El Meiche’s function in the plane *z*/*h* = 0.15, while for all the other shape functions as well as new proposed function, maximum values of the stresses are in the plane *z*/*h* = 0.2.

**Figure 12:**
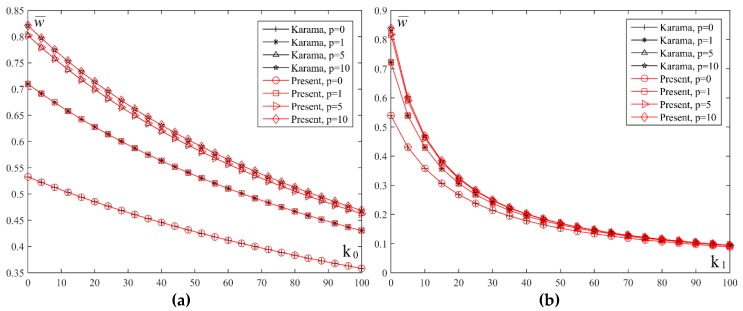
Normalized values of the displacement $\bar{w}$ of plate on elastic foundation for different values index *p* and coefficients *k*_0_ and *k*_1_: (**a**) *a*/*h* = 10, *a*/*b* = 0.2, *k*_1_ = 10; (**b**) *a*/*h* = 10, *a*/*b* = 0.2, *k*_0_ = 10.

In order to get a clear insight on the effect of Winkler and Pasternak coefficients of the elastic foundation, Figure 12 shows the diagram of the normalized values of the displacement $\bar{w}$ plate on the elastic foundation for different values of the index *p* and coefficients *k*_0_ and *k*_1._ By comparing the two diagrams, it can be seen that the change of the displacement value $\bar{w}$ is higher with the increase of the coefficient *k*_1_ value than with the increase of the coefficient *k*_0_. For example, for the FGM plate when *p* = 5, and the increase of the coefficient from *k*_0_ = 0 to *k*_0_ = 100, the value of deflection changes twice its value. In the other case, with the change of the coefficient from *k*_1_ = 0 to *k*_1_ = 100, the value of deflection changes 8 times its value.

## 7. Conclusions

The results obtained in the previously published papers have been a starting point for developing and applying the new shape function. They have emphasized the importance and topicality of the research on the application of the functionally graded materials. A thorough and comprehensive systematization and investigation of the literature on the matter have been conducted according to the problem type which authors tried to solve during FGM plate analysis. Special attention and focus have been given to different deformation theories which authors had used in their analyses. The new shape function has been presented along with the comparative review of it with 13 different shape functions which were primarily developed by different authors for the analysis of composite laminates but, in this paper, they have been adjusted and implemented in appropriate relations for the analysis of FGM plates. Based on the obtained results of the static analysis of moderately thick and thick plates, it can be concluded that the newly developed shape function could be applied in the analysis of FGM plates.

By analyzing the obtained results, the following could be concluded:

- the values of the vertical displacement $\bar{w}$ (deflection) and the corresponding stresses, which were obtained in this paper by using HSDT theory based on the new shape function, match the results of the same values obtained in the reference papers by using TSDT theory [58], quasi 3D theory of elasticity [59] and HSDT theories based on 13 different shape functions. However, in contrast to that, there are significant deviations of the results obtained for the values of the vertical displacement, especially for stresses ${\bar{\sigma }}_{xx}$, from the results obtained by CPT theory from the reference papers [60].
- the diagram of the distribution of transverse shear stresses ${\bar{\tau }}_{xz}$ and ${\bar{\tau }}_{yz}$ across the thickness of the plate shows the difference in behavior between a homogenous, ceramic or metal, plate and FGM plate. A basic property of FGM can be clearly seen, and that is the asymmetry of the stress distribution in relation to the middle plane of the plate (*z* = 0). The maximum values of stresses, depending on the volume fraction of certain constituents, are shifted in relation to the plane *z*=0, which represents a neutral plane in homogenous plates.
- the highest values of the displacement $\bar{w}$ are obtained in a metal plate, the lowest in a ceramic plate and in an FGM plate, the values are somewhere in between and they depend on the volume fraction of the constituents. Based on that, it can be concluded that by varying the volume fraction of metal and ceramic, a desired bending rigidity of the plate can be achieved.
- a comparative analysis of the change of transverse shear stresses ${\bar{\tau }}_{xz}$ and ${\bar{\tau }}_{yz}$ across the thickness of the plate shows that, unlike the stress ${\bar{\tau }}_{xy}$, their values do not match for all the shape functions.
- by introducing FG plate on Winkler–Pasternak model of elastic foundation is shown that the influence of the Winkler coefficient (*k*_0_) is smaller than the influence of the Pasternak coefficient (*k*_1_).

## Figures and Tables

**Table 1:** Shear deformation shape functions.

Number of Shape Function (SF)	Names of Authors	Shape Function *f*(*z*)
SF 1	Ambartsumain [52]	$\left(z/2\right)\left(h^{2}/4-z^{2}/3\right)$
SF 2	Kaczkowski, Panc and Reissner [53]	$\left(5z/4\right)\left(1-4z^{2}/3h^{2}\right)$
SF 3	Levy, Stein, Touratier [54]	(h/π)sin(πz/h)
SF 4	Mantari at al. [55]	$\sin\left(\pi z/h\right)e^{\cos\left(\pi z/h\right)/2}+\left(\pi z/2h\right)$
SF 5–6	Mantari at al. [45]	$\tan\left(mz\right)-zm\sec^{2}\left(mh/2\right),\;m=\left\{1/5h,\pi /2h\right\}$
SF 7	Karama at al. [56], Aydogdu [44]	$z\exp\left(-2{\left(z/h\right)}^{2}\right),z\exp\left(-2{\left(z/h\right)}^{2}/\ln\alpha \right),\;\forall \alpha >0$
SF 8	Mantari at al. [46]	$z\cdot 2.85^{-2{\left(z/h\right)}^{2}}+0.028z$
SF 9	El Meiche at al. [47]	ξ[(h/π)sin(πz/h)−z],ξ={1,1/cosh(π/2)−1}
SF 10	Soldatos [43]	hsinh(z/h)−zcosh(1/2)
SF 11	Akavci and Tanrikulu [49]	$z\sec h\left(z^{2}/h^{2}\right)-z\sec h\left(\pi /4\right)\left[1-\left(\pi /2\right)\tanh\left(\pi /4\right)\right]$
SF 12	Akavci and Tanrikulu [49]	$\left(3\pi /2\right)h\tanh\left(z/h\right)-\left(3\pi /2\right)z\sec h^{2}\left(1/2\right)$
SF 13	Mechab at al. [48]	$\frac{z\cos\left(1/2\right)}{-1+\cos\left(1/2\right)}-\frac{h\sin\left(z/h\right)}{-1+\cos\left(1/2\right)}$

**Table 2:** Material properties of FGM constituents.

Material	Material Properties	
	Elasticity Modulus, *E*[*GPa*]	Poisson’s Ratio, *ν*
Aluminum (Al)	$E_{m}=70$	ν=0.3
Alumina (Al_2_O_3_)	$E_{c}=380$	ν=0.3

**Table 3:** Normalized values of displacement and stresses of square plate for different values of the index *p* and the ratio *a*/*h* (*a*/*b* = 1). CPT: classical plate theory; TSDT: third-order shear deformation theory.

*p*	Theory	$\bar{w}$		${\bar{\sigma }}_{xx}(h/3)$		${\bar{\tau }}_{xy}(-h/3)$		${\bar{\tau }}_{xz}(h/6)$	
		*a*/*b* = 1							
		*a*/*h* = 10	*a*/*h* = 5	*a*/*h* = 10	*a*/*h* = 5	*a*/*h* = 10	*a*/*h* = 5	*a*/*h* = 10	*a*/*h* = 5
1	Present study	0.5889	0.6687	1.4899	0.7345	0.6111	0.3034	0.2604	0.2599
	CPT [60]	0.5623	-----	2.0150	-----	-----	-----	-----	-----
	Quasi 3D [59]	0.5876	-----	1.5061	-----	0.6112	-----	0.2511	-----
	TSDT [58]	0.5890	-----	1.4898	-----	0.6111	-----	0.2599	-----
	SF 1	0.5889	0.6687	1.4898	0.7344	0.6111	0.3034	0.2607	0.2602
	SF 2	0.5889	0.6687	1.4898	0.7344	0.6111	0.3034	0.2607	0.2602
	SF 3	0.5889	0.6685	1.4894	0.7336	0.6110	0.3033	0.2621	0.2615
	SF 4	0.5880	0.6648	1.4888	0.7323	0.6109	0.3030	0.2566	0.2554
	SF 5	0.5889	0.6687	1.4898	0.7344	0.6111	0.3034	0.2607	0.2601
	SF 6	0.5888	0.6683	1.4908	0.7363	0.6113	0.3038	0.2551	0.2547
	SF 7	0.5887	0.6679	1.4891	0.7330	0.6109	0.3031	0.2624	0.2616
	SF 8	0.5887	0.6679	1.4891	0.7330	0.6109	0.3031	0.2623	0.2615
	SF 9	0.5887	0.6679	1.4891	0.7330	0.6109	0.3031	0.2623	0.2615
	SF 10	0.5889	0.6687	1.4898	0.7344	0.6111	0.3034	0.2605	0.2600
	SF 11	0.5887	0.6679	1.4902	0.7352	0.6112	0.3036	0.2569	0.2566
	SF 12	0.5889	0.6686	1.4895	0.7338	0.6110	0.3033	0.2617	0.2611
	SF 13	0.5889	0.6687	1.4898	0.7343	0.6111	0.3034	0.2609	0.2603
2	Present study	0.7572	0.8670	1.3961	0.6838	0.5442	0.2696	0.2732	0.2726
	CPT [60]	-----	-----	-----	-----	-----	-----	-----	-----
	Quasi 3D [59]	0.7571	-----	1.4133	-----	0.5436	-----	0.2495	-----
	TSDT [58]	0.7573	-----	1.3960	-----	0.5442	-----	0.2721	-----
	SF 1	0.7573	0.8671	1.3960	0.6836	0.5442	0.2695	0.2736	0.2730
	SF 2	0.7573	0.8671	1.3960	0.6836	0.5442	0.2695	0.2736	0.2730
	SF 3	0.7573	0.8671	1.3954	0.6824	0.5440	0.2693	0.2763	0.2755
	SF 4	0.7563	0.8629	1.3940	0.6797	0.5437	0.2687	0.2741	0.2726
	SF 5	0.7572	0.8671	1.3961	0.6836	0.5442	0.2695	0.2735	0.2729
	SF 6	0.7568	0.8656	1.3975	0.6865	0.5444	0.2701	0.2653	0.2649
	SF 7	0.7572	0.8667	1.3949	0.6813	0.5439	0.2691	0.2777	0.2767
	SF 8	0.7572	0.8666	1.3948	0.6812	0.5439	0.2691	0.2777	0.2768
	SF 9	0.7572	0.8666	1.3948	0.6812	0.5439	0.2691	0.2777	0.2768
	SF 10	0.7572	0.8670	1.3961	0.6837	0.5442	0.2696	0.2733	0.2727
	SF 11	0.7567	0.8649	1.3969	0.6854	0.5444	0.2699	0.2667	0.2663
	SF 12	0.7573	0.8672	1.3956	0.6827	0.5441	0.2694	0.2755	0.2748
	SF 13	0.7573	0.8671	1.3960	0.6835	0.5442	0.2695	0.2739	0.2733
4	Present study	0.8814	1.0406	1.1795	0.5707	0.5669	0.2799	0.2529	0.2523
	CPT [60]	0.8281	-----	1.6049	-----	-----	-----	-----	-----
	Quasi 3D [59]	0.8823	-----	1.1841	-----	0.5671	-----	0.2362	-----
	TSDT [58]	0.8815	-----	1.1794	-----	0.5669	-----	0.2519	-----
	SF 1	0.8814	1.0409	1.1794	0.5704	0.5669	0.2798	0.2537	0.2529
	SF 2	0.8814	1.0409	1.1794	0.5704	0.5669	0.2798	0.2537	0.2529
	SF 3	0.8818	1.0423	1.1783	0.5684	0.5667	0.2795	0.2580	0.2571
	SF 4	0.8815	1.0402	1.1756	0.5630	0.5662	0.2784	0.2623	0.2606
	SF 5	0.8814	1.0408	1.1794	0.5705	0.5669	0.2799	0.2535	0.2528
	SF 6	0.8802	1.0360	1.1816	0.5749	0.5673	0.2807	0.2421	0.2417
	SF 7	0.8820	1.0429	1.1774	0.5666	0.5665	0.2791	0.2612	0.2601
	SF 8	0.8820	1.0429	1.1773	0.5664	0.5665	0.2791	0.2614	0.2603
	SF 9	0.8820	1.0429	1.1773	0.5664	0.5665	0.2791	0.2614	0.2603
	SF 10	0.8814	1.0407	1.1795	0.5706	0.5669	0.2799	0.2532	0.2525
	SF 11	0.8798	1.0346	1.1811	0.5739	0.5672	0.2805	0.2427	0.2423
	SF 12	0.8817	1.0420	1.1786	0.5690	0.5668	0.2796	0.2568	0.2559
	SF 13	0.8815	1.0411	1.1793	0.5702	0.5669	0.2798	0.2541	0.2534
8	Present study	0.9745	1.1828	0.9478	0.4544	0.5858	0.2886	0.2082	0.2076
	CPT [60]	-----	-----	-----	-----	-----	-----	-----	-----
	Quasi 3D [59]	0.9739	-----	0.9622	-----	0.5883	-----	0.2261	-----
	TSDT [58]	0.9747	-----	0.9747	-----	0.5858	-----	0.2087	-----
	SF 1	0.9746	1.1832	0.9476	0.4541	0.5858	0.2886	0.2087	0.2081
	SF 2	0.9746	1.1832	0.9476	0.4541	0.5858	0.2886	0.2087	0.2081
	SF 3	0.9749	1.1845	0.9465	0.4520	0.5856	0.2881	0.2120	0.2113
	SF 4	0.9739	1.1794	0.9435	0.4461	0.5850	0.2871	0.2139	0.2125
	SF 5	0.9745	1.1831	0.9477	0.4542	0.5858	0.2886	0.2086	0.2080
	SF 6	0.9730	1.1774	0.9500	0.4589	0.5863	0.2895	0.1995	0.1991
	SF 7	0.9751	1.1848	0.9455	0.4500	0.5854	0.2877	0.2143	0.2134
	SF 8	0.9751	1.1848	0.9454	0.4498	0.5854	0.2877	0.2145	0.2135
	SF 9	0.9751	1.1848	0.9454	0.4498	0.5854	0.2877	0.2145	0.2135
	SF 10	0.9745	1.1830	0.9477	0.4543	0.5858	0.2886	0.2084	0.2078
	SF 11	0.9727	1.1763	0.9496	0.4581	0.5861	0.2893	0.2006	0.2003
	SF 12	0.9749	1.1842	0.9469	0.4526	0.5856	0.2883	0.2111	0.2104
	SF 13	0.9746	1.1833	0.9475	0.4539	0.5858	0.2885	0.2091	0.2084
20	Present study	1.1377	1.3727	0.7710	0.3721	0.6079	0.2993	0.2011	0.2005
	CPT [60]	-----	-----	-----	-----	-----	-----	-----	-----
	Quasi 3D [59]	-----	-----	-----	-----	-----	-----	-----	-----
	TSDT [58]	-----	-----	-----	-----	-----	-----	-----	-----
	SF 1	1.1377	1.3727	0.7709	0.3720	0.6078	0.2993	0.2013	0.2008
	SF 2	1.1377	1.3727	0.7709	0.3720	0.6078	0.2993	0.2013	0.2008
	SF 3	1.1374	1.3712	0.7702	0.3707	0.6076	0.2989	0.2025	0.2019
	SF 4	1.1338	1.3561	0.7687	0.3677	0.6073	0.2982	0.1979	0.1966
	SF 5	1.1377	1.3727	0.7709	0.3720	0.6078	0.2993	0.2013	0.2007
	SF 6	1.1375	1.3723	0.7723	0.3748	0.6083	0.3002	0.1963	0.1960
	SF 7	1.1368	1.3686	0.7697	0.3696	0.6075	0.2986	0.2028	0.2019
	SF 8	1.1367	1.3683	0.7696	0.3695	0.6075	0.2986	0.2027	0.2019
	SF 9	1.1367	1.3683	0.7696	0.3695	0.6075	0.2986	0.2027	0.2019
	SF 10	1.1377	1.3727	0.7709	0.3721	0.6079	0.2993	0.2012	0.2006
	SF 11	1.1375	1.3722	0.7720	0.3741	0.6081	0.2998	0.1983	0.1979
	SF 12	1.1375	1.3718	0.7704	0.3711	0.6077	0.2990	0.2022	0.2016
	SF 13	1.1377	1.3726	0.7708	0.3718	0.6078	0.2993	0.2015	0.2009

**Table 4:** Normalized values of displacement and stresses of square plate on elastic foundation for *p* = 5, different values of the *k*_0_ и *k*_1_ and the ratio *a*/*h* (*a*/*b* = 1).

*p*	*k*_0_	*k*_1_	Theory	$\bar{w}$		${\bar{\sigma }}_{xx}$		${\bar{\tau }}_{xy}$		${\bar{\tau }}_{xz}$	
				*a*/*b* = 1							
				*a*/*h* = 10	*a*/*h* = 5	*a*/*h* = 10	*a*/*h* = 5	*a*/*h* = 10	*a*/*h* = 5	*a*/*h* = 10	*a*/*h* = 5
5	0	0	Present study	0.9113	1.0882	4.2441	2.2107	0.5757	0.2840	0.1916	0.1911
			SF 1	0.9113	1.0885	4.2447	2.2118	0.5756	0.2839	0.1929	0.1924
			SF 2	0.9113	1.0885	4.2447	2.2118	0.5756	0.2839	0.1929	0.1924
			SF 3	0.9118	1.0902	4.2488	2.2199	0.5754	0.2835	0.2016	0.2009
			SF 4	0.9115	1.0885	4.2612	2.2443	0.5748	0.2824	0.2329	0.2313
			SF 5	0.9113	1.0884	4.2445	2.2116	0.5756	0.2839	0.1927	0.1921
			SF 6	0.9098	1.0826	4.2359	2.1945	0.5761	0.2848	0.1759	0.1756
			SF 7	0.9121	1.0911	4.2527	2.2276	0.5752	0.2831	0.2104	0.2095
			SF 8	0.9121	1.0911	4.2531	2.2284	0.5752	0.2831	0.2113	0.2104
			SF 9	0.9121	1.0911	4.2531	2.2284	0.5752	0.2831	0.2113	0.2104
			SF 10	0.9112	1.0883	4.2443	2.2110	0.5756	0.2839	0.1921	0.1916
			SF 11	0.9094	1.0810	4.2359	2.1945	0.5760	0.2846	0.1668	0.1665
			SF 12	0.9117	1.0898	4.2476	2.2175	0.5755	0.2836	0.1991	0.1985
			SF 13	0.9114	1.0887	4.2450	2.2126	0.5756	0.2839	0.1937	0.1932
	100	0	Present study	0.4967	0.5450	2.3135	1.1073	0.3138	0.1422	0.1045	0.0957
			SF 1	0.4967	0.5451	2.3137	1.1076	0.3137	0.1422	0.1051	0.0963
			SF 2	0.4967	0.5451	2.3137	1.1076	0.3137	0.1422	0.1051	0.0963
			SF 3	0.4969	0.5455	2.3154	1.1108	0.3136	0.1418	0.1098	0.1005
			SF 4	0.4968	0.5451	2.3225	1.1239	0.3133	0.1414	0.1269	0.1158
			SF 5	0.4967	0.5451	2.3136	1.1076	0.3137	0.1422	0.1050	0.0962
			SF 6	0.4963	0.5436	2.3107	1.1019	0.3142	0.1430	0.0960	0.0882
			SF 7	0.4969	0.5457	2.3172	1.1142	0.3134	0.1416	0.1146	0.1048
			SF 8	0.4969	0.5457	2.3174	1.1146	0.3134	0.1416	0.1151	0.1052
			SF 9	0.4969	0.5457	2.3174	1.1146	0.3134	0.1416	0.1151	0.1052
			SF 10	0.4967	0.5450	2.3135	1.1074	0.3138	0.1422	0.1047	0.0959
			SF 11	0.4961	0.5432	2.3112	1.1028	0.3143	0.1430	0.0910	0.0837
			SF 12	0.4968	0.5454	2.3148	1.1098	0.3136	0.1419	0.1085	0.0993
			SF 13	0.4967	0.5451	2.3138	1.6370	0.3137	0.1421	0.1056	0.0967
	0	10	Present study	0.3442	0.3668	1.6032	0.7451	0.2175	0.0957	0.0724	0.0644
			SF 1	0.3442	0.3667	1.6033	0.7453	0.2174	0.0956	0.0728	0.0648
			SF 2	0.3442	0.3667	1.6033	0.7453	0.2174	0.0956	0.0728	0.0648
			SF 3	0.3443	0.3669	1.6043	0.7472	0.2172	0.0954	0.0761	0.0676
			SF 4	0.3442	0.3667	1.6093	0.7562	0.2171	0.0951	0.0879	0.0779
			SF 5	0.3442	0.3667	1.6033	0.7452	0.2174	0.0956	0.0727	0.0647
			SF 6	0.3440	0.3661	1.6017	0.7421	0.2178	0.0963	0.0665	0.0594
			SF 7	0.3443	0.3670	1.6055	0.7494	0.2171	0.0952	0.0794	0.0704
			SF 8	0.3443	0.3671	1.6057	0.7496	0.2171	0.0952	0.0797	0.0707
			SF 9	0.3443	0.3671	1.6057	0.7496	0.2171	0.0952	0.0797	0.0707
			SF 10	0.3442	0.3667	1.6032	0.7451	0.2174	0.0957	0.0725	0.0645
			SF 11	0.3439	0.3659	1.6022	0.7428	0.2178	0.0963	0.0631	0.0563
			SF 12	0.3442	0.3669	1.6040	0.7466	0.2173	0.0955	0.0752	0.0668
			SF 13	0.3442	0.3668	1.6034	1.2283	0.2174	0.0956	0.0731	0.0650
	100	10	Present study	0.2617	0.2745	1.2190	0.5578	0.1654	0.0716	0.0550	0.0482
			SF 1	0.2617	0.2745	1.2190	0.5579	0.1653	0.0716	0.0554	0.0485
			SF 2	0.2617	0.2745	1.2190	0.5579	0.1653	0.0716	0.0554	0.0485
			SF 3	0.2617	0.2746	1.2197	0.5592	0.1652	0.0714	0.0578	0.0506
			SF 4	0.2617	0.2745	1.2236	0.5661	0.1650	0.0712	0.0668	0.0583
			SF 5	0.2617	0.2745	1.2190	0.5661	0.1653	0.0716	0.0553	0.0484
			SF 6	0.2616	0.2741	1.2180	0.5578	0.1656	0.0721	0.0506	0.0444
			SF 7	0.2617	0.2747	1.2206	0.5608	0.1651	0.0712	0.0604	0.0527
			SF 8	0.2617	0.2747	1.2207	0.5610	0.1651	0.0712	0.0606	0.0529
			SF 9	0.2617	0.2747	1.2207	0.5610	0.1651	0.0712	0.0606	0.0529
			SF 10	0.2617	0.2745	1.2190	0.5578	0.1653	0.0716	0.0551	0.0483
			SF 11	0.2615	0.2740	1.2184	0.5564	0.1656	0.0721	0.0479	0.0422
			SF 12	0.2617	0.2746	1.2195	0.5588	0.1652	0.0714	0.0571	0.0500
			SF 13	0.2617	0.2745	1.2191	0.9722	0.1653	0.0716	0.0556	0.0487

**Table 5:** Normalized values of displacement and stresses of square plate on elastic foundation for *p* = 10, different values of the *k*_0_ и *k*_1_ and the ratio *a*/*h* (*a*/*b* = 1).

*p*	*k*_0_	*k*_1_	Theory	$\bar{w}$		${\bar{\sigma }}_{xx}$		${\bar{\tau }}_{xy}$		${\bar{\tau }}_{xz}$	
				*a*/*b* =1							
				*a*/*h* = 10	*a*/*h* = 5	*a*/*h* = 10	*a*/*h* = 5	*a*/*h* = 10	*a*/*h* = 5	*a*/*h* = 10	*a*/*h* = 5
10	0	0	Present study	1.0086	1.2273	5.0843	2.6423	0.5896	0.2904	0.2101	0.2095
			SF 1	1.0087	1.2275	5.0848	2.6434	0.5895	0.2903	0.2113	0.2107
			SF 2	1.0087	1.2275	5.0848	2.6434	0.5895	0.2903	0.2113	0.2107
			SF 3	1.0089	1.2282	5.0890	2.6515	0.5893	0.2899	0.2198	0.2190
			SF 4	1.0071	1.2201	5.1006	2.6742	0.5888	0.2889	0.2488	0.2472
			SF 5	1.0086	1.2275	5.0847	2.6431	0.5895	0.2903	0.2111	0.2104
			SF 6	1.0074	1.2229	5.0758	2.6255	0.5900	0.2913	0.1944	0.1940
			SF 7	1.0088	1.2277	5.0928	2.6590	0.5891	0.2895	0.2281	0.2272
			SF 8	1.0088	1.2275	5.0931	2.6597	0.5891	0.2895	0.2290	0.2280
			SF 9	1.0088	1.2275	5.0931	2.6597	0.5891	0.2895	0.2290	0.2280
			SF 10	1.0086	1.2274	5.0845	2.6426	0.5896	0.2903	0.2105	0.2099
			SF 11	1.0072	1.2222	5.0762	2.6263	0.5899	0.2910	0.1852	0.1849
			SF 12	1.0088	1.2281	5.0877	2.6491	0.5894	0.2900	0.2174	0.2166
			SF 13	1.0087	1.2276	5.0852	2.6442	0.5895	0.2903	0.2121	0.2115
	100	0	Present study	0.5243	0.5779	2.6430	1.2440	0.3065	0.1367	0.1092	0.0986
			SF 1	0.5243	0.5779	2.6432	1.2444	0.3064	0.1366	0.1098	0.0992
			SF 2	0.5243	0.5779	2.6432	1.2444	0.3064	0.1366	0.1098	0.0992
			SF 3	0.5244	0.5780	2.6451	1.2479	0.3063	0.1364	0.1142	0.1030
			SF 4	0.5239	0.5762	2.6534	1.2630	0.3063	0.1364	0.1294	0.1167
			SF 5	0.5243	0.5779	2.6432	1.2443	0.3064	0.1367	0.1097	0.0990
			SF 6	0.5240	0.5768	2.6401	1.2385	0.3069	0.1374	0.1011	0.0915
			SF 7	0.5243	0.5779	2.6471	1.2517	0.3062	0.1363	0.1186	0.1069
			SF 8	0.5243	0.5779	2.6474	1.2521	0.3062	0.1363	0.1190	0.1073
			SF 9	0.5243	0.5779	2.6474	1.2521	0.3062	0.1363	0.1190	0.1073
			SF 10	0.5243	0.5778	2.6431	1.2441	0.3064	0.1367	0.1094	0.0988
			SF 11	0.5239	0.5767	2.6405	1.2392	0.3068	0.1373	0.0963	0.0872
			SF 12	0.5243	0.5780	2.6445	1.2468	0.3063	0.1365	0.1130	0.1019
			SF 13	0.5243	0.5779	2.6434	1.2447	0.3064	0.1366	0.1102	0.0995
	0	10	Present study	0.3573	0.3813	1.8008	0.8209	0.2088	0.0902	0.0744	0.0651
			SF 1	0.3572	0.3813	1.8010	0.8212	0.2088	0.0902	0.0748	0.0654
			SF 2	0.3572	0.3813	1.8010	0.8212	0.2088	0.0902	0.0748	0.0654
			SF 3	0.3572	0.3814	1.8022	0.8234	0.2087	0.0900	0.0778	0.0680
			SF 4	0.3570	0.3806	1.8084	0.8342	0.2087	0.0901	0.0882	0.0771
			SF 5	0.3572	0.3813	1.8009	0.8211	0.2088	0.0902	0.0747	0.0653
			SF 6	0.3571	0.3809	1.7992	0.8177	0.2091	0.0907	0.0689	0.0604
			SF 7	0.3572	0.3813	1.8036	0.8259	0.2086	0.0899	0.0808	0.0705
			SF 8	0.3572	0.3813	1.8038	0.8262	0.2086	0.0899	0.0811	0.0708
			SF 9	0.3572	0.3813	1.8038	0.8262	0.2086	0.0899	0.0811	0.0708
			SF 10	0.3572	0.3813	1.8009	0.8210	0.2088	0.0902	0.0745	0.0652
			SF 11	0.3570	0.3808	1.7995	0.8183	0.2091	0.0906	0.0656	0.0576
			SF 12	0.3572	0.3814	1.8018	0.8227	0.2087	0.0900	0.0770	0.0672
			SF 13	0.3572	0.3813	1.8011	0.9376	0.2088	0.0901	0.0751	0.0657
	100	10	Present study	0.2692	0.2826	1.3569	0.6084	0.1574	0.0669	0.0561	0.0482
			SF 1	0.2691	0.2826	1.3570	0.6086	0.1573	0.0668	0.0564	0.0485
			SF 2	0.2691	0.2826	1.3570	0.6086	0.1573	0.0668	0.0564	0.0485
			SF 3	0.2692	0.2826	1.3579	0.6102	0.1572	0.0667	0.0586	0.0504
			SF 4	0.2690	0.2822	1.3628	0.6186	0.1573	0.0668	0.0664	0.0571
			SF 5	0.2691	0.2826	1.3570	0.6086	0.1573	0.0668	0.0563	0.0484
			SF 6	0.2691	0.2823	1.3558	0.6062	0.1576	0.0672	0.0519	0.0448
			SF 7	0.2692	0.2826	1.3590	0.6121	0.1572	0.0666	0.0608	0.0523
			SF 8	0.2692	0.2826	1.3591	0.6123	0.1572	0.0666	0.0611	0.0525
			SF 9	0.2692	0.2826	1.3591	0.6123	0.1572	0.0666	0.0611	0.0525
			SF 10	0.2691	0.2826	1.3569	0.6085	0.1573	0.0668	0.0562	0.0483
			SF 11	0.2690	0.2823	1.3561	0.6067	0.1575	0.0672	0.0494	0.0427
			SF 12	0.2692	0.2826	1.3576	0.6097	0.1572	0.0667	0.0580	0.0498
			SF 13	0.2692	0.2826	1.3571	0.6087	0.1573	0.0668	0.0566	0.0486
